# Novel Genes Affecting Blood Pressure Detected Via Gene-Based Association Analysis

**DOI:** 10.1534/g3.115.016915

**Published:** 2015-03-26

**Authors:** Huan Zhang, Xing-Bo Mo, Tan Xu, Xiao-Qing Bu, Shu-Feng Lei, Yong-Hong Zhang

**Affiliations:** * Jiangsu Key Laboratory of Preventive and Translational Medicine for Geriatric Diseases, Soochow University, Suzhou, Jiangsu, 215123, People’s Republic of China; † Department of Epidemiology, School of Public Health, Medical College of Soochow University, Suzhou, Jiangsu, People’s Republic of China; ‡ Center for Genetic Epidemiology and Genomics, School of Public Health, Medical College of Soochow University, Suzhou, Jiangsu, 215123, People’s Republic of China

**Keywords:** genome-wide association study, gene-based association, blood pressure, coronary artery disease, protein–protein interaction

## Abstract

Hypertension is a common disorder and one of the most important risk factors for cardiovascular diseases. The aim of this study was to identify more novel genes for blood pressure. Based on the publically available SNP-based *P* values of a meta-analysis of genome-wide association studies, we performed an initial gene-based association study in a total of 69,395 individuals. To find supplementary evidence to support the importance of the identified genes, we performed GRAIL (gene relationships among implicated loci) analysis, protein–protein interaction analysis, functional annotation clustering analysis, coronary artery disease association analysis, and other bioinformatics analyses. Approximately 22,129 genes on the human genome were analyzed for blood pressure in gene-based association analysis. A total of 43 genes were statistically significant after Bonferroni correction (*P* < 2.3×10^−6^). The evidence obtained from the analyses of this study suggested the importance of *ID1* (*P* = 2.0×10^−6^), *CYP17A1* (*P* = 4.58×10^−9^), *ATXN2* (*P* = 1.07×10^−13^), *CLCN6* (*P* = 4.79×10^−9^), *FURIN* (*P* = 1.38×10^−6^), *HECTD4* (*P* = 3.95×10^−11^), *NPPA* (*P* = 1.60×10^−6^), and *PTPN11* (*P* = 8.89×10^−10^) in the genetic basis of blood pressure. The present study found some important genes associated with blood pressure, which might provide insights into the genetic architecture of hypertension.

Hypertension is a common disorder and one of the most important risk factors for cardiovascular diseases (CVD), the leading cause of death worldwide (Kearney *et al.* 2005). Blood pressure (BP) is influenced by both lifestyle and genetic factors (Whelton *et al.* 2002). Identification of genes predisposing to hypertension will increase our understanding of the genetic mechanisms and provide the framework to identify potential novel drug targets for the treatment of hypertension and prevention of CVD.

Genetic factors contribute to the variation of BP, with heritability estimates of approximately 40–60% (Kupper *et al.* 2005). A previous large-scale meta-analysis of genome-wide association studies (GWAS) has identified 29 BP-associated loci. However, the total variance explained by the 29 discovered signals was (only) 0.94% for diastolic blood pressure (DBP) and 0.92% for systolic blood pressure (SBP) (Ehret *et al.* 2011). This means that many more genetic factors need to be identified.

Because gene-based association analysis method can combine genetic information given by all the single nucleotide polymorphisms (SNP) in a gene, it can increase the capability of finding novel genes and obtain more informative results. Gene-based association method has several attractive features. For example, it can substantially reduce the burden of multiple-testing correction, and the extension of the findings to further functional analyses is more straightforward. This method has been used as a novel method complementing SNP-based GWAS to identify disease susceptibility genes (Li *et al.* 2011).

Based on the publically available datasets, this study presented a statistically robust gene-based association analysis, focusing on finding more relevant genes for BP. Further, we performed gene relationships among implicated loci (GRAIL) analysis, protein–protein interaction (PPI) analysis, functional annotation clustering analysis, coronary artery disease (CAD) association analysis, and other bioinformatics analyses to find supplementary information for the identified genes.

## Materials and Methods

### Gene-based association analysis

The present gene-based association study used data from the International Consortium for Blood Pressure Genome-Wide Association Studies (ICBP GWAS) (Ehret *et al.* 2011). Raw data were the downloaded association *P* values of approximately 2.5 million SNPs from the initial SNP-based GWAS for SBP and DBP. Study design, subject characteristics, genotyping, data-quality filters, and SNP-based association analyses were detailed in the original GWAS meta-analysis publication (Ehret *et al.* 2011). Briefly, it was a meta-analysis of GWAS-evaluated associations between 2.5 million genotyped or imputed SNPs and BP in 69,395 individuals of European ancestry from 29 studies.

Gene-based association analysis was performed using the GATES (Gene-based Association Test using Extended Simes procedure) method, which was modeled in KGG software, a systematic biological knowledge-based mining system for genome-wide genetic studies (Li *et al.* 2011). The extended Simes test integrated functional information and association evidence to combine the *P* values of the SNPs within a gene to obtain an overall *P* value for the association of the entire gene. This test was powerful and did not require the raw genotype or phenotype data as inputs. It offered effective control of the type 1 error rate regardless of gene size and linkage disequilibrium (LD) pattern among markers, and did not need permutation or simulation to evaluate empirical significance. In the present gene-based association analysis, data files (for SBP and DBP association analyses) each containing four input variables (the rs number, chromosome, position, and SNP-based association *P* value) for KGG were prepared using the R program. The defined length of the extended gene region was from 2-kb upstream to 2-kb downstream of each gene. LD was adjusted based on CEU genotype data from HapMap release 2 in the analyses. Bonferroni correction (Tarone 1990), the simplest and most conservative approach, was used to adjust for multiple testing in the analyses.

### Text-mining-based data analysis

To examine the relationship among these genes in genomic BP regions, we performed a GRAIL analysis (http://www.broadinstitute.org/mpg/grail/) (Raychaudhuri *et al.* 2009). GRAIL is a text-mining tool that identifies nonrandom, evidence-based links between genes using PubMed abstracts. GRAIL gives a score to each region, which is a statistical significance score that reflects the degree of relatedness among genes at different loci. The inputs were the most associated SNPs in each gene revealed by the gene-based analyses and other BP-associated SNPs reported in previous GWAS (Ehret *et al.* 2011; Lu *et al.* 2015). The GRAIL analysis parameters were set as follows: genome assembly, release 22/HG18; HapMap population, CEU; functional datasource, PubMed text (December 2006); gene-size correction, on; gene list, all human genes within database; and default settings for SNP rs number submission and all SNPs as query and seed. Setting the PubMed text to December 2006 can avoid the confounding of the high number of GWAS publications. We used the VIZ-GRAIL software (http://www.broadinstitute.org/mpg/grail/vizgrail.html) (Raychaudhuri 2011), which was implemented as two separate perl scripts, to visualize the results from the GRAIL analysis with default parameters.

### Protein–protein interaction network

To obtain functional evidence for the identified BP-associated genes, we searched for PPI networks using the STRING database (http://string-db.org/) and the Disease Association Protein–Protein Link Evaluator (Dapple) algorithm. STRING is a database of known and predicted protein interactions. The interactions include direct (physical) and indirect (functional) associations derived from genomic context, high-throughput experiments, coexpression, and previous knowledge (text mining) (Franceschini *et al.* 2013). A list of 75 BP-associated genes was uploaded for the PPI analysis in STRING. Dapple searched for significant physical connectivity among proteins encoded by genes in loci associated with disease according to PPIs reported in the literatures and revealed highly connected genes by testing the significance of biological networks using a permutation method (1000 permutations) (Rossin *et al.* 2011). The input for Dapple was a list of 30 SNPs representing the BP-associated regions, and the list of 75 genes was uploaded as seed genes.

### Functional annotation clustering analysis

To gain insights into the functions of the identified genes, we tested the probability of the identified genes clustering into a specific Gene Ontology (GO) term or a particular biological pathway. The functional annotation clustering analysis was performed by using the Database for Annotation, Visualization, and Integrated Discovery (DAVID) (http://david.abcc.ncifcrf.gov/) (Huang da *et al.* 2009a,b). A list of 75 BP-associated genes was analyzed. The default annotation categories were selected in the functional annotation clustering analysis. The enrichment could be quantitatively measured by using a Fisher exact test. Bonferroni correction (Tarone 1990) was used to adjust for multiple testing in the analysis.

### Association with coronary artery disease

Because hypertension is one of the most important risk factors for CAD, we evaluated the associations between CAD and the BP-associated genes identified in the gene-based association analysis. Raw data were the downloaded *P* values from a large-scale GWAS meta-analysis for CAD performed by the CARDIoGRAM and C4D consortium (Deloukas *et al.* 2013). This dataset was publically available at ftp://ftp.sanger.ac.uk/pub/cardiogramplusc4d/. This study comprised 63,746 CAD cases and 130,681 controls. Some of these samples were used in the ICBP GWAS discovery analysis, including 2287 cases and 22,024 controls from the CHARGE consortium, 1121 controls from MIGen, 1473 controls from B58C, and 795 controls from PROCARDIS. The gene-based associations were tested using the GATES method described above. We used the Bonferroni method to adjust for multiple testing in this gene-based association analysis (Tarone 1990).

### Gene-trait association score

Prior knowledge can provide additional evidence for prioritizing genes. We used Online Mendelian Inheritance in Man (OMIM; http://www.ncbi.nlm.nih.gov/omim/) to determine whether the detected genes were involved in monogenic syndromes. In addition, we used Mouse Genome Informatics (MGI; http://www.informatics.jax.org/) to determine if the identified genes were involved in phenotypes in mice and other mammals. To find out whether any of the identified BP-associated genes had been reported in previous association studies of complex diseases, we searched the database of HuGE Integrator (http://www.hugenavigator.net/HuGENavigator/) for information. Then, we constructed a simple gene-trait association score as evidence for prioritizing the detected genes, according to the following items: *P* < 0.05 or the gene is the best candidate in GRAIL analysis; candidate genes for complex diseases according to HuGE; evidence from OMIM, MGI, STRING, Dapple, and DAVID; and CAD association.

## Results

### Gene-based association analysis

**Figure 1:**
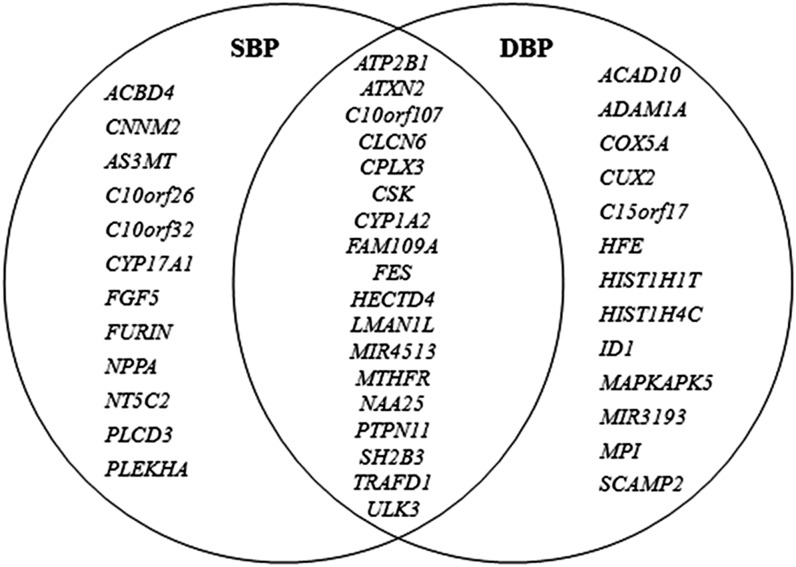
Venn diagram of pleiotropic associations of the BP-associated genes. Forty-three genes that achieved the gene-based test significance level of 2.3×10^−6^ are included. Among them, 30 were associated with SBP, 31 were associated with DBP, and 18 genes overlap.

In the gene-based association analysis, approximately 1,248,073 (48.7%) SNPs were mapped onto 22,129 genes on the human genome for SBP and DBP. The QQ plots of genes and SNPs were shown in the Supporting Information, Figure S1 and Figure S2. According to the Bonferroni correction method, the significance level for the gene-based tests was 2.3×10^−6^ for each BP measure. Accordingly, 43 significant genes located in 14 loci were found for BP. Among them, 30 were associated with SBP (Table 1), 31 were associated with DBP (Table 2), with 18 genes overlap (Figure 1). Within several of these 43 genes, the most associated variants did not reach the genome-wide significance threshold of 5.0×10^−8^ in the original SNP-based GWAS, for example, rs5068 (1.86×10^−7^) in *NPPA*, rs3742004 (1.01×10^−6^) in *FAM109A*, rs11854147 (7.16×10^−7^) in *CYP1A2*, rs2521501 (1.22×10^−7^ for SBP and 6.06×10^−7^ for DBP) in *FES*, rs8032315 (3.02×10^−7^) in *FURIN*, rs198851 (2.45×10^−7^) in *HIST1H4C*, rs659964 (3.65×10^−7^) in *ACAD10*, rs4766705 (2.05×10^−6^) in *ADAM1A*, rs3213628 (2.09×10^−6^) in *MAPKAPK5*, and rs6058197 (2.0×10^−6^) in *ID1* (and *MIR3193*). Several genes possessed genome-wide significant SNPs, but they were not reported or evaluated in previous GWAS. These genes included *C10orf32*, *WBP1L* (*C10orf26*), *NAA25*, *TRAFD1*, *MIR4513*, *CUX2*, *COX5A*, *FAM219B* (*C15orf17*), *MPI*, and *SCAMP2*. These 43 BP-associated genes were located in 14 regions, and 13 of these regions were previously reported. Only one region, 20q11.21 (*ID1* and *MIR3193*), had not been confirmed by ICBP GWAS (Figure S3, Figure S4, Figure S5, Figure S6, Figure S7, Figure S8, Figure S9, and Figure S10).

**Table 1:** Association results for SBP-associated genes with gene-based *P* < 2.3×10^−6^

Gene	*P*_Gene	Locus		SNP*^b^*			*P*_CAD	Score
		Position	Reported*^a^*	ID	Feature	*P*_SNP		
*CLCN6*	4.79E-09	1p36	*NPPB*	rs17367504	intronic	2.11E-10		5
*NPPA*	1.60E-06	1p36.21	*—*	rs5068	3UTR	1.86E-07		8
*MTHFR*	2.74E-09	1p36.3	*MTHFR*	rs17367504	intronic	2.11E-10		7
*FGF5*	2.18E-07	4q21	*FGF5*	rs16998073	upstream	2.84E-08		5
*C10orf107*	1.44E-06	10q21.2	*C10orf107*	rs4590817	intronic	5.95E-08		2
*NT5C2*	4.27E-09	10q24.32	*NT5C2*	rs11191548	downstream	5.03E-10	5.10E-08	4
*CYP17A1*	4.58E-09	10q24.32	—	rs1004467	intronic	6.61E-10	3.13E-08	8
*AS3MT*	5.89E-09	10q24.32	—	rs11191454	intronic	1.12E-09	7.02E-09	5
*C10orf32*	5.89E-09	10q24.32	—	rs3824754	intronic	9.75E-10	7.83E-09	2
*CNNM2*	5.39E-09	10q24.32	—	rs1926032	intronic	5.72E-10	6.71E-09	4
*WBP1L*	1.26E-07	10q24.32	—	rs486955	intronic	9.47E-09		2
*PLEKHA7*	3.64E-07	11p15.1	*PLEKHA7*	rs381815	intronic	2.45E-09		3
*ATP2B1*	2.62E-11	12q21.3	*ATP2B1*	rs2681472	intronic	1.32E-12		5
*SH2B3*	7.71E-09	12q24	*SH2B3*	rs3184504	exonic	1.69E-09	8.10E-11	7
*ATXN2*	6.07E-09	12q24	—	rs653178	intronic	9.3E-10	3.52E-08	7
*FAM109A*	2.02E-06	12q24	—	rs3742004	3UTR	1.01E-06	4.09E-06	2
*HECTD4*	2.01E-07	12q24	—	rs11066188	intronic	1.72E-08	1.63E-07	4
*NAA25*	4.12E-07	12q24	—	rs17696736	intronic	3.43E-08	1.88E-07	3
*PTPN11*	5.97E-07	12q24	—	rs11066320	intronic	4.56E-08	1.82E-07	10
*TRAFD1*	8.62E-08	12q24	—	rs17630235	downstream	1.45E-08	1.41E-08	4
*ULK3*	2.41E-07	15q24.1	*ULK3*	rs6495122	downstream	6.03E-08		3
*CPLX3*	1.09E-07	15q24.1	—	rs7162232	intronic	2.33E-08		5
*CSK*	2.05E-09	15q24.1	—	rs1378942	intronic	3.43E-10		4
*MIR4513*	1.07E-09	15q24.1	—	rs1378942	intronic	3.43E-10		1
*CYP1A2*	1.93E-06	15q24.1	—	rs11854147	downstream	7.16E-07		5
*LMAN1L*	4.23E-08	15q24.1	—	rs7176022	intronic	2.11E-08		2
*FES*	4.40E-07	15q26.1	*FES*	rs2521501	intronic	1.22E-07		5
*FURIN*	1.38E-06	15q26.1	*FURIN*	rs8032315	intronic	3.02E-07	5.35E-10	6
*ACBD4*	5.59E-07	17q21.31	*ZNF652*	rs12946454	intronic	8.91E-08		1
*PLCD3*	8.11E-07	17q21.31	—	rs12946454	intronic	8.91E-08		4

a Genes reported in this region by the ICBP GWAS.

b The most associated SNP within the corresponding gene identified by gene-based analysis.

**Table 2:** Association results for DBP-associated genes with gene-based *P* < 2.3×10^−6^

Gene	*P*_Gene	Locus		SNP*^b^*			*P*_CAD	Score
		Position	Reported*^a^*	ID	Feature	*P*_SNP		
*CLCN6*	1.24E-07	1p36	*NPPB*	rs12567136	intronic	1.15E-08		5
*MTHFR*	1.42E-07	1p36.3	*MTHFR*	rs17367504	intronic	1.29E-08		7
*HFE*	4.06E-07	6p22.1	*HFE*	rs1799945	exonic	4.78E-08		7
*HIST1H1T*	2.25E-07	6p22.1	—	rs198846	downstream	3.80E-08		3
*HIST1H4C*	1.46E-06	6p22.1	—	rs198851	downstream	2.45E-07		3
*C10orf107*	4.14E-08	10q21.2	*C10orf107*	rs4590817	intronic	1.77E-09		2
*ATP2B1*	7.74E-08	12q21.3	*ATP2B1*	rs2681472	intronic	3.90E-09		5
*SH2B3*	1.05E-13	12q24	*SH2B3*	rs3184504	exonic	2.33E-14	8.10E-11	7
*ACAD10*	1.60E-06	12q24	—	rs659964	intronic	3.65E-07		1
*ATXN2*	1.07E-13	12q24	—	rs653178	intronic	1.64E-14	3.52E-08	7
*CUX2*	6.21E-07	12q24	—	rs7306529	intronic	4.87E-08	8.15E-07	4
*FAM109A*	4.30E-08	12q24	—	rs3742004	3UTR	2.15E-08	4.09E-06	2
*ADAM1A*	2.06E-06	12q24	—	rs4766705	intergenic	2.05E-06		3
*HECTD4*	3.95E-11	12q24	—	rs11066188	intronic	3.06E-12	1.63E-07	4
*MAPKAPK5*	2.09E-06	12q24	—	rs3213628	intronic	2.09E-06	2.34E-06	3
*NAA25*	1.97E-10	12q24	—	rs17696736	intronic	2.80E-11	1.88E-07	3
*PTPN11*	8.89E-10	12q24	—	rs11066320	intronic	6.32E-11	1.82E-07	10
*TRAFD1*	1.42E-11	12q24	—	rs17630235	downstream	2.92E-12	1.41E-08	4
*ULK3*	4.90E-08	15q24.1	*ULK3*	rs12487	upstream	1.31E-08		3
*COX5A*	7.86E-08	15q24.1	—	rs1133323	downstream	4.52E-08		4
*CPLX3*	1.64E-09	15q24.1	—	rs6495122	downstream	8.41E-10		5
*CSK*	2.07E-11	15q24.1	—	rs1378942	intronic	3.47E-12		4
*CYP1A2*	2.48E-09	15q24.1	—	rs2470890	exonic	1.03E-09		5
*C15orf17*	8.39E-08	15q24.1	—	rs11856413	upstream	7.64E-08		0
*LMAN1L*	3.17E-09	15q24.1	—	rs7162232	intronic	1.58E-09		2
*MIR4513*	1.96E-11	15q24.1	—	rs1378941	intronic	7.38E-12		1
*MPI*	8.38E-08	15q24.1	—	rs7495739	intronic	5.02E-08		4
*SCAMP2*	2.80E-08	15q24.1	—	rs3765066	intronic	4.17E-09		3
*FES*	1.69E-06	15q26.1	*FES*	rs2521501	intronic	6.06E-07		5
*ID1*	2.0E-06	20q11.21	—	rs6058197	upstream	2.0E-06		5
*MIR3193*	2.0E-06	20q11.21	—	rs6058197	upstream	2.0E-06		1

a Genes reported in this region by the ICBP GWAS.

b The most associated SNP within the corresponding gene identified by gene-based analysis.

### GRAIL analysis

**Figure 2:**
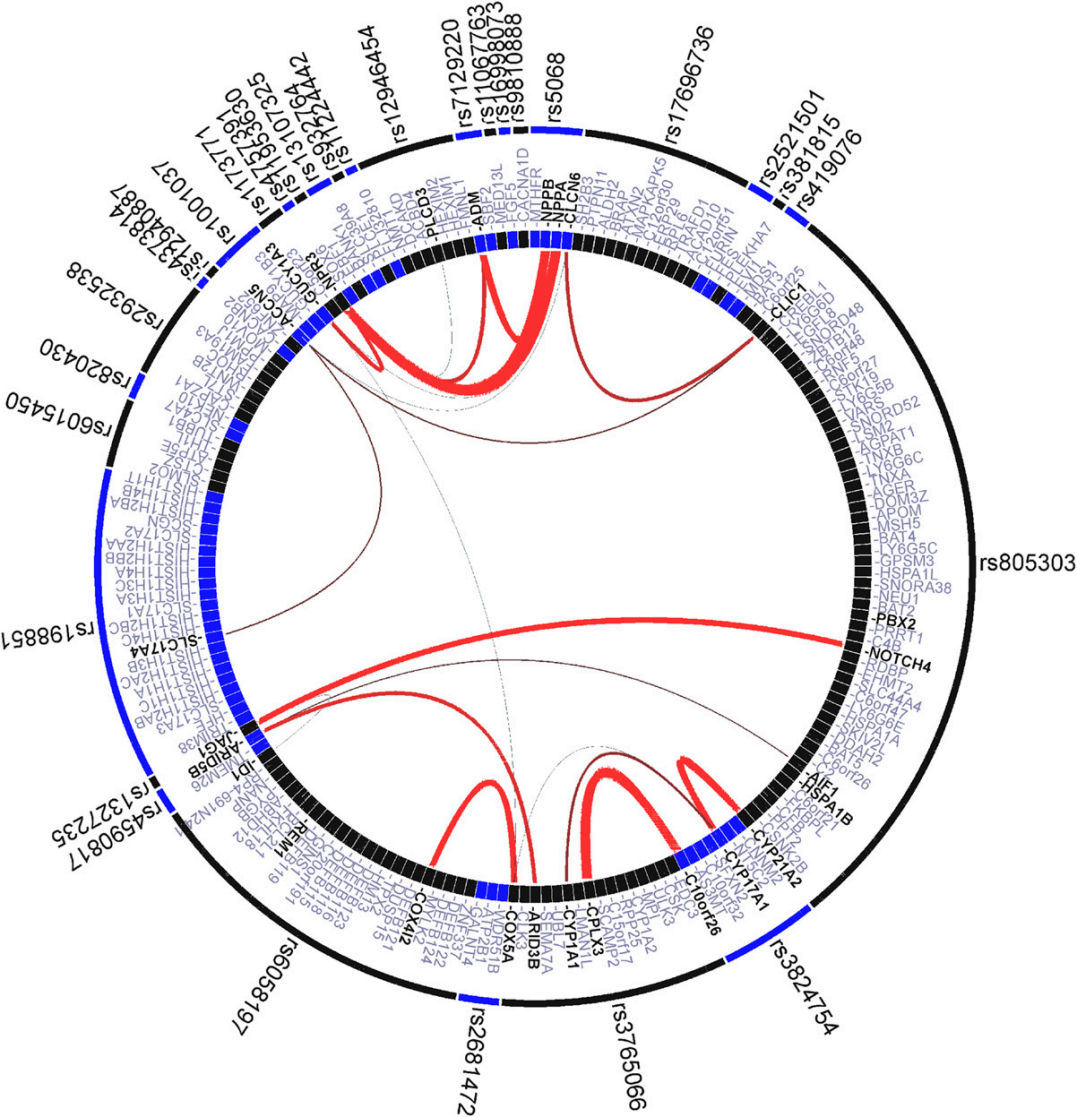
GRAIL output results visualized with the VIZ-GRAIL software. Genes in the 30 BP loci are shown in the circle. The lines between genes represent individually significant connections that contributed to the positive signal, with the thickness of the lines being inversely proportional to the probability that a literature-based connection would be seen by chance. Gene names indicated in bold are listed if individual *P* value for the connection has a *P*__GRAIL_ < 0.05.

We applied the GRAIL text-mining algorithm to investigate connections between genes tagged in the 30 BP loci. GRAIL identified 20 key words that were commonly associated with the BP candidate genes in the literatures, for example, "natriuretic," "notch," "calcium," "cotransporter," "cyclase," "differentiation," "atrial," "voltage," "pump," "cardiac," "smooth," and "vascular." A total of 190 candidate genes were found by GRAIL analysis. The analysis demonstrated that several of these loci were functionally related. It revealed significant (*P*_GRAIL_ < 0.05) connections between 26 genes of the 30 input loci (Figure 2). The connection results highlighted notable biological functions for sets of genes within BP-associated regions. Three of the unreported genes, *ID1*, *WBP1L* (*C10orf26*), and *COX5A* (gene-based *P* < 2.3×10^−6^), had a high degree of connectivity with genes in other associated loci: *ID1* shared similar functions with *JAG1*; *WBP1L* (*C10orf26*) shared similar functions with *CPLX3*; and *COX5A* shared similar functions with *COX412*.

### Protein–protein interactions

**Figure 3:**
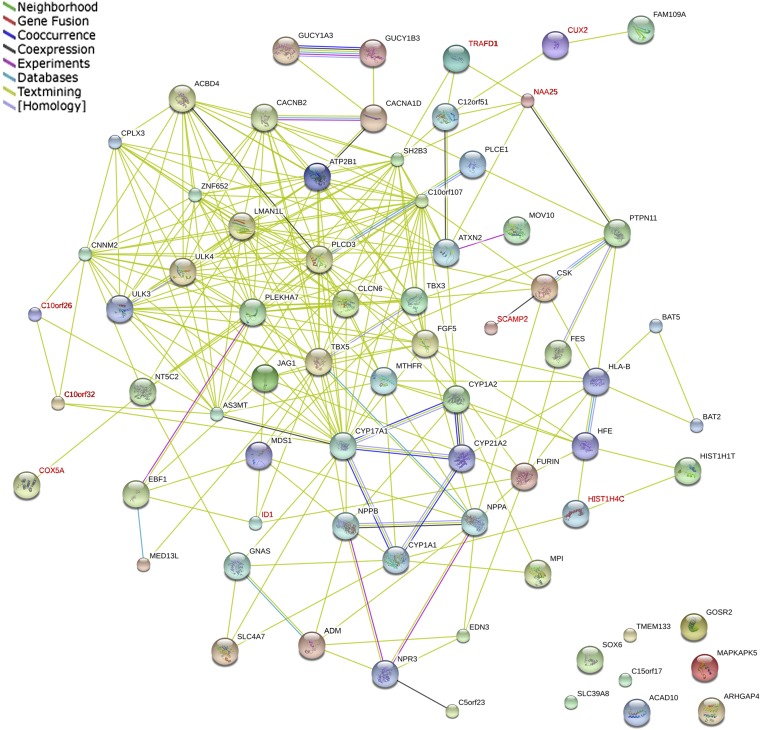
Protein–protein interactions between BP-associated genes in STRING. Red gene symbols indicate genes identified in the present gene-based association study (nine genes), whereas black indicates previously reported genes.

**Figure 4:**
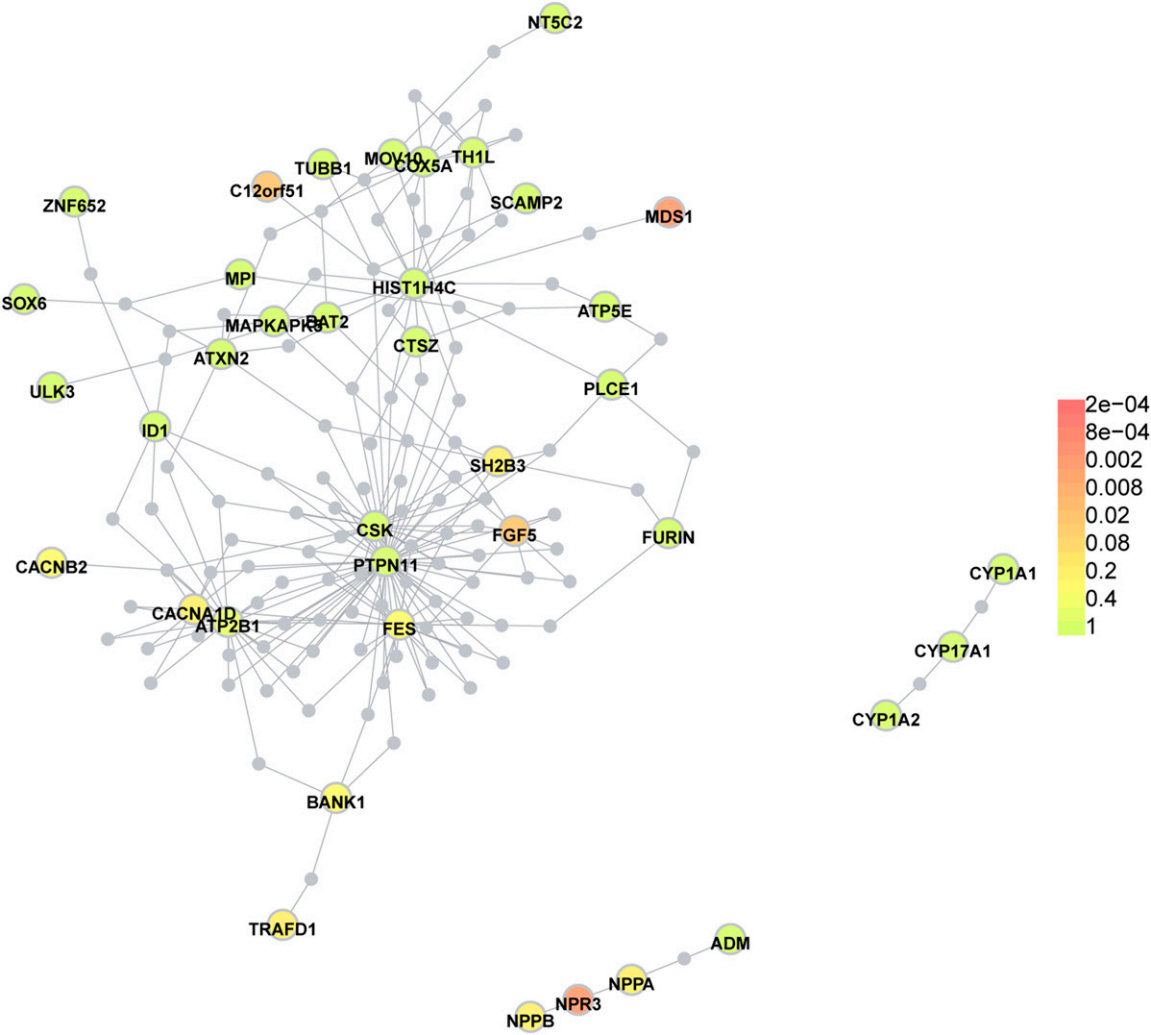
Protein–protein interactions between BP-associated genes in Dapple. The three groups of direct interactions and common interactors between indirect connections are presented. Gray circles are common interactors.

We detected PPIs between 75 BP genes in the STRING database. Most of these genes showed connections with each other, except *ACAD10*, *ARHGAP4*, *C150rf17*, *MAPKAPK5*, *GOSR2*, *SLC39A8*, *SOX6*, and *TMEM133* (Figure 3). Interaction evidence for 9 of the 16 unreported BP-associated genes, namely *TRAFD1*, *CUX2*, *NAA25*, *SCAMP2*, *WBP1L* (*C10orf26*), *C10orf32*, *COX5A*, *ID1*, and *HIST1H4C* were detected. We considered two categories of interactions in Dapple PPI networks: direct (between the BP-associated genes themselves) and indirect (via common interactors). The connected proteins formed three groups (Figure 4). Twenty of the 43 detected genes were presented in the network. Dapple identified seven disease proteins participating in four direct connections: *COX5A* with *TH1L*, *PTPN11* with *CSK*, *NPR3* with *NPPA*, and *NPR3* with *NPPB*. For the 75 seed proteins, we identified four significant for connectivity: *MDS1* (rs419076, *P* = 2.0×10^−3^); *NPR3* (rs1173771, *P* = 2.0×10^−3^); *C12orf51* (rs17696736, *P* = 1.19×10^−2^); and *FGF5* (rs16998073, *P* = 2.38×10^−2^) (all *P* values corrected).

### Functional annotation clustering analysis

The BP genes tend to enrich in the regulation of the blood circulation, circulatory system process, toxin metabolic process, and *trans*-Golgi network GO terms, and in heme and transmembrane protein SP_PIR_KEYWORDS categories (Table 3). Twelve BP-associated genes were involved in these categories. We still focused on finding unreported genes in these categories, and two genes were found. They were *SCAMP2* *in trans*-Golgi network GO term (GO: 0005802) and *COX5A* in heme SP_PIR_KEYWORDS categories.

**Table 3:** Results of functional annotation clustering analysis for the BP-associated genes

Category	Term	Count	%	Genes	Fold Enrichment	*P*	Bonferroni
GOTERM_BP_FAT	GO:0008015∼blood circulation	8	1.24	*EDN3*, *MTHFR*, *ADM*, *GUCY1A3*, *NPPB*, *GUCY1B3*, *NPR3*, *NPPA*	10.77	7.50E-06	0.006
GOTERM_BP_FAT	GO:0003013∼circulatory system process	8	1.24	*EDN3*, *MTHFR*, *ADM*, *GUCY1A3*, *NPPB*, *GUCY1B3*, *NPR3*, *NPPA*	10.77	7.50E-06	0.006
GOTERM_BP_FAT	GO:0009404∼toxin metabolic process	3	0.47	*CYP1A1*, *AS3MT*, *CYP1A2*	150.31	1.49E-04	0.047
GOTERM_CC_FAT	GO:0005802∼*trans*-Golgi network	4	0.62	*ATXN2*, ***SCAMP2***, *GNAS*, *FURIN*	24.70	5.21E-04	0.056
SP_PIR_KEYWORDS	heme	6	0.93	*CYP17A1*, *CYP1A1*, *CYP21A2*, *GUCY1B3*, *CYP1A2*, ***COX5A***	14.24	5.96E-05	0.005
SP_PIR_KEYWORDS	transmembrane protein	10	1.55	*ATP2B1*, *CYP17A1*, *CYP1A1*, *CYP21A2*, *HLA-B*, *CYP1A2*, *NPR3*, *CLCN6*, *FURIN*, *CACNA1D*	4.47	3.29E-04	0.011

### Association with coronary artery disease

To view whether the 43 BP-associated genes were associated with CAD, we performed gene-based analysis for CAD association using the published GWAS dataset. In this analysis, 45,010 (55.4%) SNPs were mapped onto 10,303 genes on the human genome. The significance level for this gene-based test was 4.85×10^−6^. We found that 15 of the 43 BP-associated genes seemed to be associated with CAD (Table 1 and Table 2). Among these CAD-associated genes, *CYP17A1*, *AS3MT*, *CNNM2*, *NT5C2*, *ATXN2*, *FAM109A*, *SH2B3*, *HECTD4*, *PTPN11*, and *FURIN* were previously reported BP-associated genes, whereas *C10orf32*, *NAA25*, *TRAFD1*, *CUX2*, and *MAPKAPK5* were not.

### Gene-trait association score

The genetic association information collected from the HuGE Navigator for the 43 detected genes was summarized in Table S1. Several genes have not been reported as candidate genes for complex diseases, including *ACBD4*, *C10orf32*, *C15orf17*, *COX5A*, *FAM109A*, *ID1*, *MAPKAPK5*, *MPI*, *SCAMP2*, and *TRAFD1*. Many of the detected genes have been reported to be associated with CAD, type 2 diabetes, hypertension, and stroke. The information collected from the OMIM and MGI databases for the 43 genes was summarized in Table S2. Nineteen genes showed no information in OMIM or MGI databases. Nine genes were involved in monogenic syndromes, including *ATXN2*, *CNNM2*, *CYP17A1*, *HFE*, *MPI*, *MTHFR*, *NPPA*, *PTPN11*, and *SH2B3*. Five genes had a knock-out mouse presenting with human defects, including *ATXN2*, *CLCN6*, *HFE*, *MTHFR*, and *PTPN11*. Aggregating the results of the association analyses and evidence derived from GRAIL, STRING, Dapple, DAVID, HuGE, OMIM, and MGI databases, we constructed a gene-trait association score as evidence for prioritizing the detected genes. The *PTPN11* gene got the highest score, and the *NPPA* and *CYP17A1* gene followed. *ATXN2*, *MTHFR*, *HFE*, and *SH2B3* also scored high (Table 1, Table 2, and Table S3). Taken together, eight genes (*ATXN2*, *CLCN6*, *CYP17A1*, *FURIN*, *HECTD4*, *ID1*, *NPPA*, and *PTPN11*) obtained evidence from at least four of the following five items: GRAIL analysis; PPI analysis (STRING and Dapple); functional annotation clustering analysis (DAVID); evidence from databases (OMIM, MGI, HuGE); and CAD association analysis.

## Discussion

The present gene-based association study identified 43 BP-associated genes. Bioinformatics analyses and CAD association analysis provided supportive evidence and functional information on the association of several of these genes with BP, *e.g.*, *ATXN2*, *CLCN6*, *CYP17A1*, *FURIN*, *HECTD4*, *ID1*, *NPPA*, and *PTPN11*.

During the past 8 years, GWAS have revolutionized the understanding of the genetic architecture of complex diseases and traits. Twenty-nine loci that influenced BP variation have been confirmed in a large-scale GWAS meta-analysis (Ehret *et al.* 2011). Other than this study, more genes or loci associated with BP or hypertension have been reported. These findings have provided important insights into the biology and mechanisms of hypertension.

Because millions of SNPs are tested in GWAS, keeping the significance threshold at the conventional value of 0.05 would lead to a large number of false-positive results. This multiple testing burden has led to the adoption of stringent significance thresholds in GWAS (Sham and Purcell 2014). However, with stringent significance thresholds adopted, many moderate association signals in GWAS datasets would be ignored. However, GWAS always focused on the most significant variations when replicating the associations, and reported genes according to physical locations of the genome. In regions that contain dozens of genes in large stretches of LD, many important genes would be missed. In the present study, we used a gene-based association analysis method to complementarily analyze the published data of GWAS (Ehret *et al.* 2011). As expected, we identified several important genes associated with BP.

Some well-known genes, such as *ATXN2*, *CLCN6*, *CYP17A1*, *FURIN*, *NPPA*, and *PTPN11*, were detected in the gene-based association analysis. *ATXN2* was involved in the Parkinson’s disease pathway. It acts as a negative regulator of endocytic EGFR internalization at the plasma membrane. Diseases associated with *ATXN2* included cardiovascular disease (Ikram *et al.* 2010), thrombotic antiphospholipid syndrome (Ochoa *et al.* 2013), and amyotrophic lateral sclerosis (Lahut *et al.* 2012), and others. *PTPN11* was located in the same region with *ATXN2*. It was involved in various pathways, such as heart development, EGFR1 signaling pathway, insulin signaling, regulation of IFNA signaling, and interferon alpha/beta signaling, and others. It is associated with several monogenic disorders, including LEOPARD syndrome 1 (LEOPARD1) (MIM: 151100), Noonan syndrome 1 (NS1) (MIM: 163950), Juvenile myelomonocytic leukemia (JMML) (MIM: 607785), and metachondromatosis (MC) (MIM: 156250). *CLCN6*, *CYP17A1*, *FURIN*, and *NPPA* were important BP-associated genes, which have been discussed in the original ICBP GWAS (Ehret *et al.* 2011). The evidence taken together from the present gene-based association analyses and bioinformatics analyses also supported the importance of these genes in the genetic basis of BP.

*ID1* located in 20q11.21 has not been reported in ICBP GWAS, but the best SNP rs6058197 (*P* = 2.0×10^−6^) in the gene was close to the GWAS significance threshold. *ID1* is an effector of the p53-dependent DNA damage response pathway (Qian and Chen 2008). The protein encoded by this gene may play a role in cell growth, senescence, and differentiation. Pathways related to this gene include heart development, TGF-beta signaling pathway, Id signaling pathway, immune response IL-3 activation and signaling pathway, Rap1 signaling pathway, and others. In the present study, we found supportive evidence for this gene from GRAIL analysis, PPI analysis, functional annotation clustering analysis, and MGI. However, the genetic association between this gene and BP as well as other traits or diseases was unclear. *MIR3193* was a noncoding gene located in 20q11.21 and shared the same signal of rs6058197 with *ID1*. Little was known about this noncoding gene. Further studies of the relationship between these genes and BP should be suggested.

The function and disease association of the *HECTD4* gene were not so clear, but it may function as E3 ubiquitin-protein ligase, which accepts ubiquitin from an E2 ubiquitin-conjugating enzyme in the form of a thioester and then directly transfers the ubiquitin to targeted substrates. The present study has suggested the importance of this gene in the etiology of hypertension. It may be an important candidate gene for hypertension.

This gene-based association study has some potential limitations. First, there were a number of gene-based analysis methods, but we only used the GATES method, without considering other methods in this study. The GATES method is powerful and more suitable for analyzing summary results of larger-scale GWAS meta-analysis. Second, we wanted to validate the results of the gene-based analysis, but no suitable dataset was found for this purpose. Datasets from two GWAS (Yang *et al.* 2012; Kelly *et al.* 2013) were analyzed, but only the significance of *C10orf32* was detected in data reported by Kelly *et al.* (2013). However, the ICBP GWAS data should be reliable because they were from a study with a large sample size and a reasonable analysis. Except 20q11.21 (*ID1* and *MIR3193*), the 13 loci detected in the present gene-based study have been confirmed by ICBP GWAS. Although we found supplementary functional information to support the significant findings, further studies were needed to validate the associations and elucidate the mechanisms.

In conclusion, the present study took advantage of the gene-based association method to perform a supplementary analysis of the GWAS dataset and found some important BP-associated genes. A series of bioinformatics analyses gave supportive evidence for the gene-based association analysis discoveries. Our findings may provide insights into the genetic basis of hypertension.

## Supplementary Material

Supporting Information
