# Supplementary material for: Novel Genes Affecting Blood Pressure Detected Via Gene-Based Association Analysis
Source: G3 (Bethesda). 2015 Mar 26;5(6):1035–42. doi: 10.1534/g3.115.016915 (PMC4478534; doi:10.1534/g3.115.016915)
Supplement: Supporting Information [file supp_5_6_1035__index.html]

Novel Genes Affecting Blood Pressure Detected Via Gene-Based Association Analysis — Supporting Information 

# Novel Genes Affecting Blood Pressure Detected Via Gene-Based Association Analysis

## Supporting Information for Zhang *et al.*, 2015

**Files in this Data Supplement:**

- Supporting Information - Figures S1-S10 and Tables S1-S3 (PDF, 1 MB)
- Figure S1 - QQ plot of genes and SNPs for DBP (ICBP GWAS data). (PDF, 524 KB)
- Figure S2 - QQ plot of genes and SNPs for SBP (ICBP GWAS data). (PDF, 525 KB)
- Figure S3 - Regional association plot for DBP at 12q24.12 (ICBP GWAS data) *CUX2*, *ACAD10* were unreported genes in this region with gene-based P value < 2.3×10^-6^. (PDF, 607 KB)
- Figure S4 - Regional association plot for DBP at 12q24.13 (ICBP GWAS data) *ADAM1A, MAPKAPK5, NAA25, TRAFD1* were unreported genes in this region with gene-based P value < 2.3×10^-6^. (PDF, 606 KB)
- Figure S5 - Regional association plot for DBP at 6p22.1 (ICBP GWAS data) *HIST1H4C* was unreported genes in this region with gene-based P value < 2.3×10^-6^. (PDF, 600 KB)
- Figure S6 - Regional association plot for DBP at 20q11.21 (ICBP GWAS data) *ID1, MIR3193* were unreported genes in this region with gene-based P value < 2.3×10^-6^. (PDF, 598 KB)
- Figure S7 - Regional association plot for DBP at 15q24.1 (ICBP GWAS data) *COX5A, C15orf17, MIR4513, SCAMP2* were unreported genes in this region with gene-based P value < 2.3×10^-6^. (PDF, 537 KB)
- Figure S8 - Regional association plot for SBP at 10q24.32 (ICBP GWAS data) *C10orf32, C10orf26 (WBP1L)* were unreported genes in this region with gene-based P value < 2.3×10^-6^. (PDF, 547 KB)
- Figure S9 - Regional association plot for SBP at 15q24.1 (ICBP GWAS data *MIR4513* were unreported genes in this region with gene-based P value < 2.3×10^-6^. (PDF, 536 KB)
- Figure S10 - Regional association plot for SBP at 12q24.13 (ICBP GWAS data) *NAA25, TRAFD1* were unreported genes in this region with gene-based P value < 2.3×10^-6^. (PDF, 537 KB)
- Table S1 - Information for the BP-associated genes collected from the HuGE Navigator. (PDF, 487 KB)
- Table S2 - Information of monogenic syndromes and mouse models for BP-associated genes. (PDF, 454 KB)
- Table S3 - Score construction for BP-associated genes. (PDF, 495 KB)
