# Supplementary material for: Novel Genes Affecting Blood Pressure Detected Via Gene-Based Association Analysis
Source: G3 (Bethesda). 2015 Mar 26;5(6):1035–42. doi: 10.1534/g3.115.016915 (PMC4478534; doi:10.1534/g3.115.016915)
Supplement: Supporting Information [file supp_g3.115.016915_016915SI.pdf]

G3·Genes | Genomes | Genetics

Novel genes affecting blood pressure detected via gene-based association analysis

Huan Zhang[§,†,#], Xing-Bo Mo[§,†,‡,#], Tan Xu[§, †], Xiao-Qing Bu[§, †], Shu-Feng Lei[§, †,‡], Yong-Hong Zhang[§, †,*]

[§].Jiangsu Key Laboratory of Preventive and Translational Medicine for Geriatric Diseases, Soochow University, Suzhou, Jiangsu, P. R. China

[†].Department of Epidemiology, School of Public Health, Medical College of Soochow University, Suzhou, Jiangsu, P. R. China

[‡].Center for Genetic Epidemiology and Genomics, School of Public Health, Medical College of Soochow University, Suzhou, Jiangsu, P. R. China

[#] These authors contributed equally to this work.

*Corresponding author: Yong-Hong Zhang, Ph.D. Address: 199 Renai Road, Suzhou Industrial Park, Suzhou, Jiangsu 215123, P. R. China

Email: yhzhang@suda.edu.cn. Tel/FAX: +86-512-65882625.

DOI: 10.1534/g3.115.016915

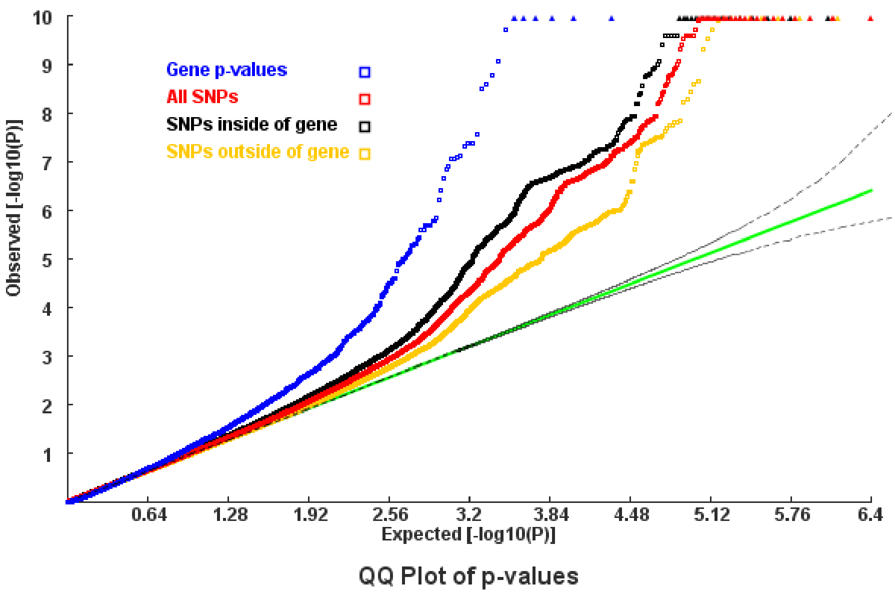

Figure S1 QQ plot of genes and SNPs for DBP (ICBP GWAS data).

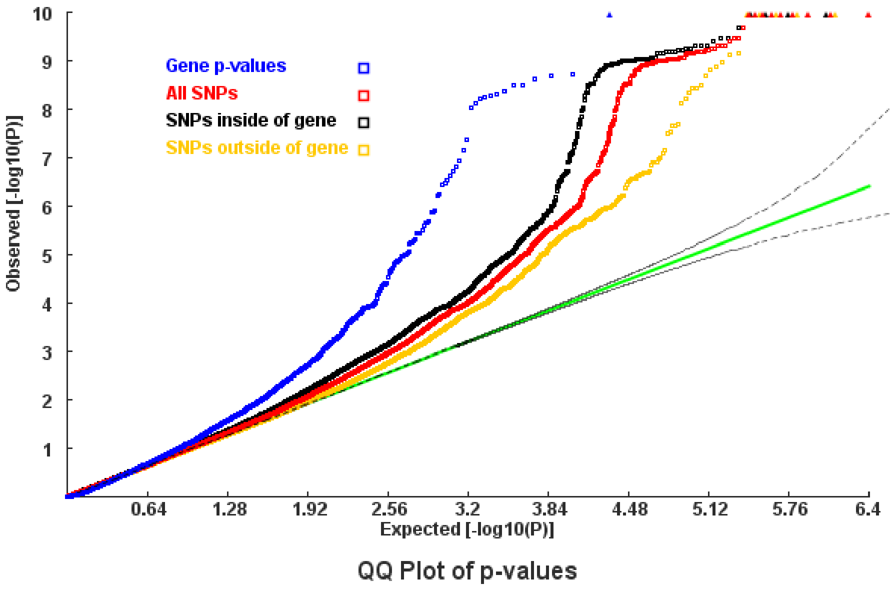

Figure S2 QQ plot of genes and SNPs for SBP (ICBP GWAS data).

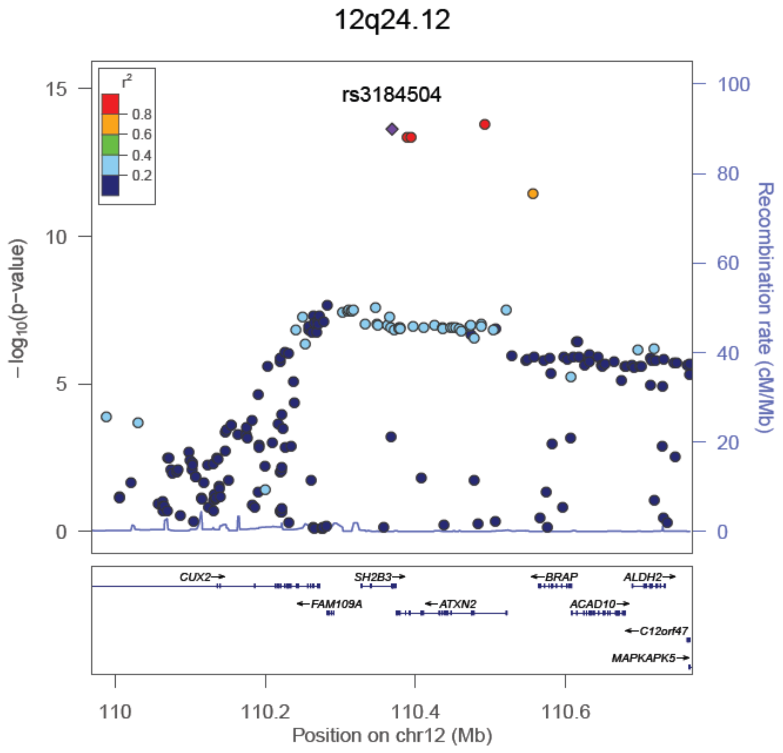

Figure S3 Regional association plot for DBP at 12q24.12 (ICBP GWAS data)

CUX2, *ACAD10* were unreported genes in this region with gene-based P value < 2.3×10^{-6}.

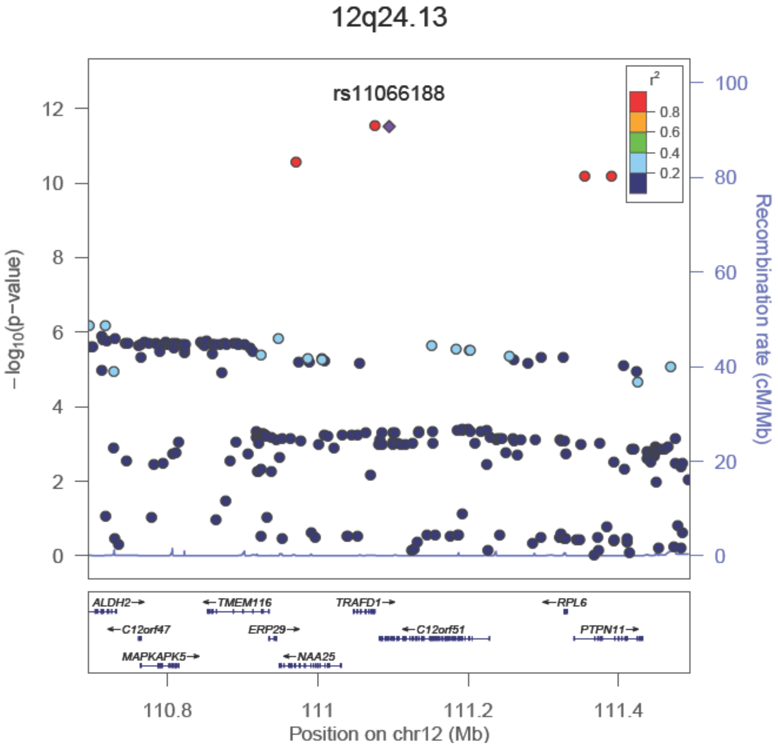

Figure S4 Regional association plot for DBP at 12q24.13 (ICBP GWAS data)

ADAM1A, *MAPKAPK5*, *NAA25*, *TRAFD1* were unreported genes in this region with gene-based P value < 2.3×10^{-6}.

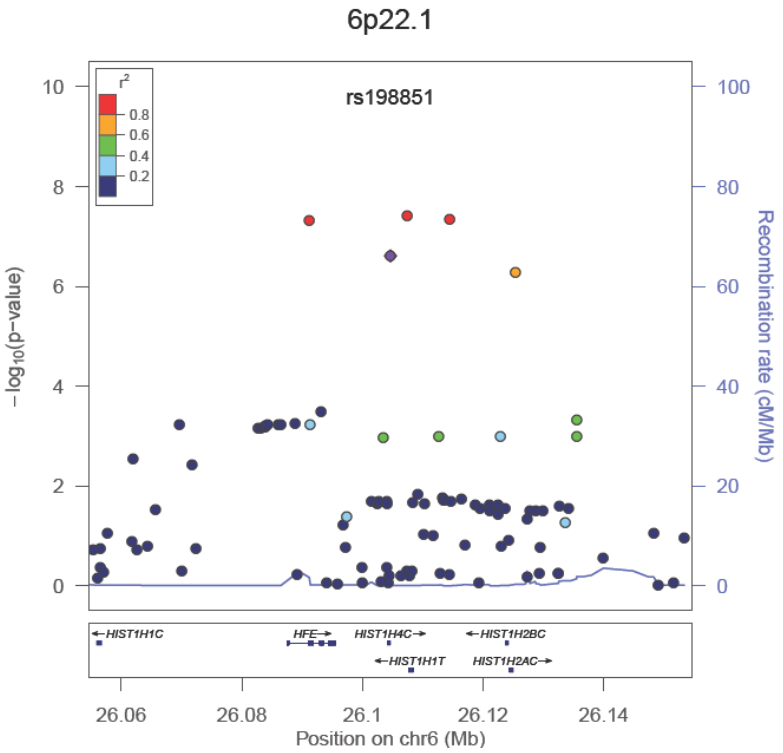

Figure S5 Regional association plot for DBP at 6p22.1 (ICBP GWAS data)

HIST1H4C was unreported genes in this region with gene-based P value < 2.3×10^{-6}.

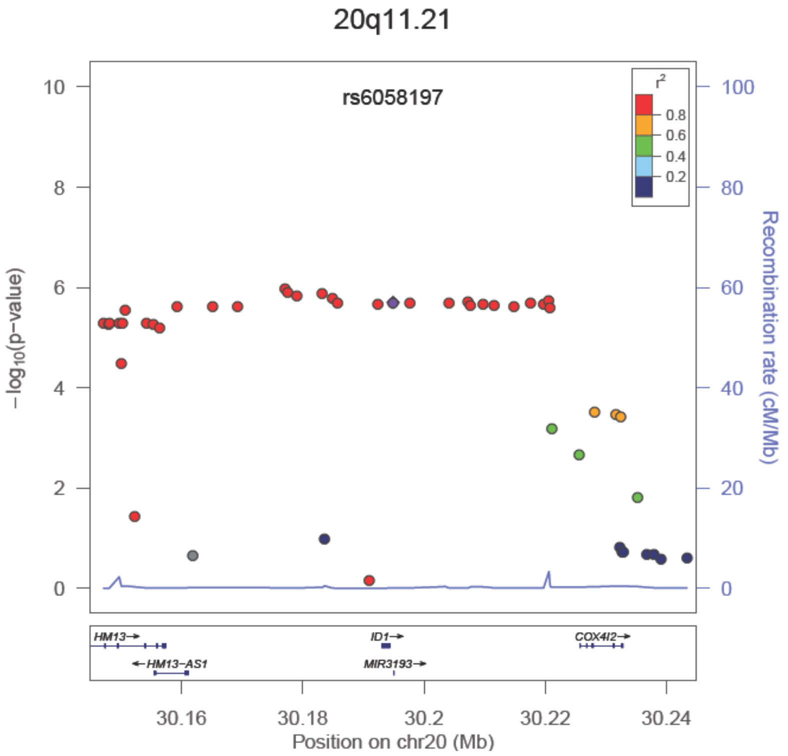

Figure S6 Regional association plot for DBP at 20q11.21 (ICBP GWAS data)

ID1, *MIR3193* were unreported genes in this region with gene-based P value < 2.3×10^{-6}.

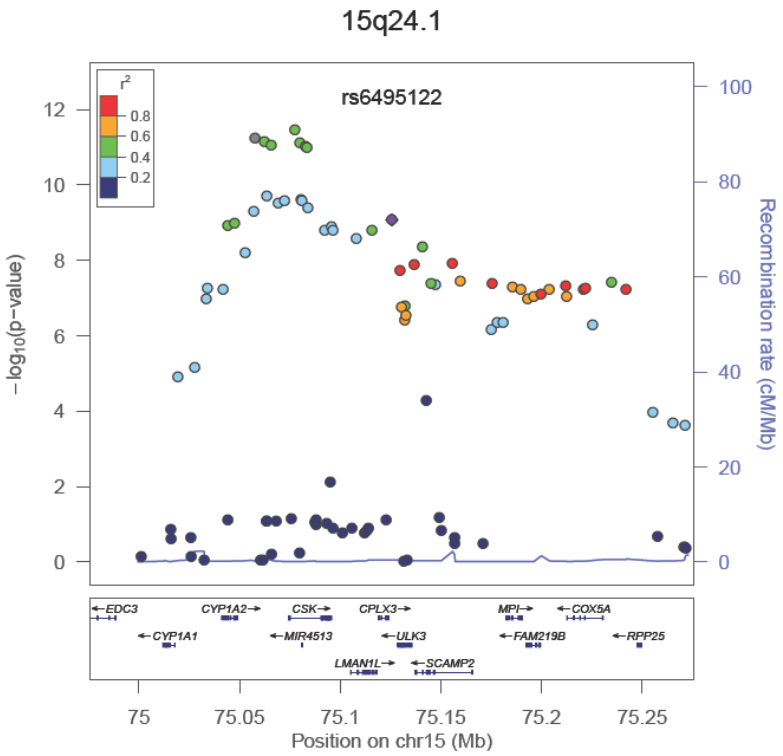

Figure S7 Regional association plot for DBP at 15q24.1 (ICBP GWAS data)

COX5A, *C15orf17*, *MIR4513*, *SCAMP2* were unreported genes in this region with gene-based P value $< 2.3\times10^{-6}$.

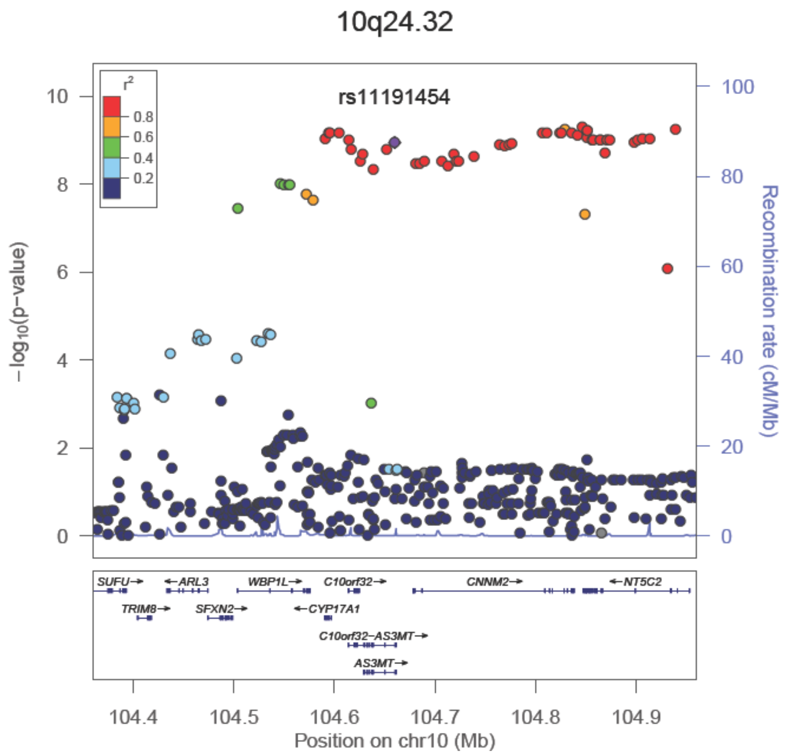

Figure S8 Regional association plot for SBP at 10q24.32 (ICBP GWAS data)

C10orf32, *C10orf26 (WBP1L)* were unreported genes in this region with gene-based P value < 2.3×10^{-6}.

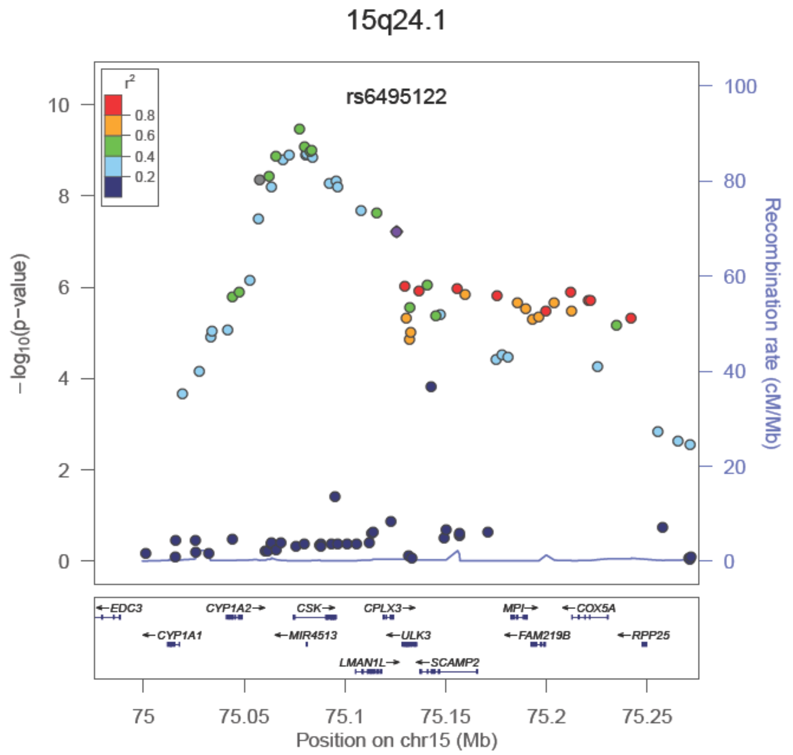

Figure S9 Regional association plot for SBP at 15q24.1 (ICBP GWAS data)

MIR4513 were unreported genes in this region with gene-based P value < 2.3×10^{-6}.

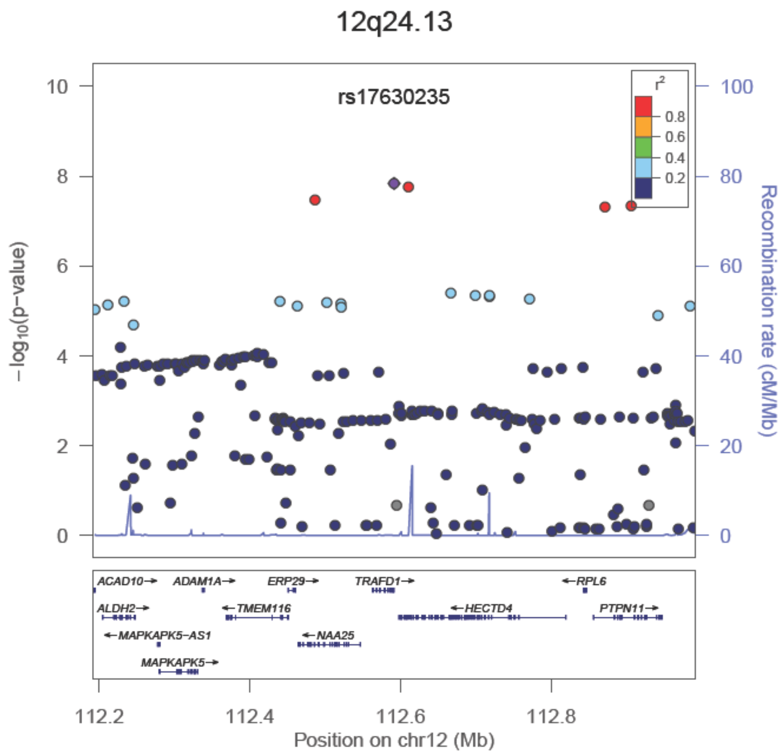

Figure S10 Regional association plot for SBP at 12q24.13 (ICBP GWAS data)

NAA25, *TRAFD1* were unreported genes in this region with gene-based P value < 2.3×10^{-6}.

Table S1 Information for the BP-associated genes collected from the HuGE Navigator

Gene_Symbol	Abortion	AIDS	Adenocarcinoma	Alcoholism	Alzheimer Disease	Amyotrophic Lateral Sclerosis	Anemia	Arsenic Poisoning Phenopedia	Arthritis, Rheumatoid	Bipolar Disorder	Breast Neoplasms	Cardiovascular Diseases	Celiac Disease	cocaine dependence	Colorectal Neoplasms	COPD	Crohn Disease	Diabetes Mellitus, Type 1
ACAD10		Y									Y							
ACBD4																		
ADAM1A																		Y
AS3MT								Y				Y						Y
ATP2B1												Y						
ATXN2						Y						Y						
C10orf107					Y							Y						
C10orf32																		
C15orf17																		
CLCN6												Y						
CNNM2					Y							Y						
COX5A																		
CPLX3														Y				
CSK												Y						
CUX2																	Y	Y
CYP17A1			Y		Y						Y	Y						
CYP1A2			Y								Y	Y			Y	Y		
FAM109A																		
FES												Y						
FGF5				Y								Y						
FURIN												Y						
HECTD4																		
HFE					Y	Y						Y						
HIST1H1T	Y																	
HIST1H4C	Y																	
ID1																		
LMAN1L																		
MAPKAPK5																		
MIR3193																		
MIR4513																		
MPI																		
MTHFR	Y			Y	Y		Y		Y		Y	Y			Y		Y	
NAA25									Y	Y		Y					Y	Y
NPPA												Y						
NT5C2												Y						
PLCD3																		
PLEKHA7																		
PTPN11																		
SCAMP2																		
SH2B3												Y	Y					Y
TRAFD1																		
ULK3																		
WBP1L					Y													

Gene_Symbol	Diabetes Mellitus, Type 2	Diabetic Nephropathies	Disease Progression	Down Syndrome	Dyslipidemias	Endometrial Neoplasms	Folic Acid Deficiency	Gout	Graves' disease	Hemochromatosis	Hepatitis	Hyperhomocysteinemia	Hypertension	hypothyroidism	Insulin Resistance	Iron Overload	Kidney Failure, Chronic	Leukemia
ACAD10	Y		Y												Y			
ACBD4																		
ADAM1A		Y																
AS3MT													Y					
ATP2B1	Y				Y								Y					
ATXN2								Y					Y				Y	
C10orf107	Y												Y					
C10orf32																		
C15orf17																		
CLCN6	Y												Y					
CNNM2													Y					
COX5A																		
CPLX3																		
CSK													Y					
CUX2	Y																	
CYP17A1						Y							Y					
CYP1A2						Y												
FAM109A																		
FES													Y				Y	
FGF5													Y					
FURIN													Y				Y	
HECTD4	Y												Y					
HFE	Y									Y	Y		Y			Y		
HIST1H1T																		
HIST1H4C																		
ID1																		
LMAN1L																		
MAPKAPK5																		
MIR3193																		
MIR4513																		
MPI																		
MTHFR	Y	Y		Y	Y		Y					Y	Y		Y		Y	
NAA25	Y																	
NPPA	Y												Y					
NT5C2													Y					
PLCD3													Y					
PLEKHA7													Y					
PTPN11																		Y
SCAMP2																		
SH2B3									Y		Y		Y	Y				
TRAFD1																		
ULK3													Y					
WBP1L																		

Gene_Symbol	Liver disease	Lung Neoplasms	Metabolic Diseases	Multiple Sclerosis	Neural Tube Defects	Noonan Syndrome	Obesity	Pancreatic Neoplasms	Parkinson Disease	Prostatic Neoplasms	Schizophrenia	Spinocerebellar Ataxias	Stroke	Thrombosis	Tobacco Use Disorder	Urinary Bladder Neoplasms	Vitiligo
ACAD10															Y		
ACBD4																	
ADAM1A																	
AS3MT											Y					Y	
ATP2B1			Y				Y										
ATXN2								Y	Y			Y					
C10orf107															Y		
C10orf32																	
C15orf17																	
CLCN6													Y		Y		
CNNM2											Y		Y		Y		
COX5A																	
CPLX3																	
CSK							Y										
CUX2																	
CYP17A1		Y					Y			Y			Y		Y		
CYP1A2		Y							Y	Y	Y					Y	
FAM109A																	
FES													Y				
FGF5																	
FURIN													Y				
HECTD4			Y												Y		
HFE	Y			Y													
HIST1H1T																	
HIST1H4C																	
ID1																	
LMAN1L															Y		
MAPKAPK5																	
MIR3193																	
MIR4513																	
MPI																	
MTHFR	Y	Y	Y		Y				Y		Y		Y	Y		Y	
NAA25																	
NPPA													Y				
NT5C2											Y		Y				
PLCD3																	
PLEKHA7																	
PTPN11						Y											
SCAMP2																	
SH2B3				Y									Y				Y
TRAFD1																	
ULK3									Y								
WBP1L																	

Table S2 Information of monogenic syndromes and mouse models for BP-associated genes

**Gene_Symbol**	**OMIM**	**MGI**	**Human Diseases Modeled in Mice**
ACAD10			
ACBD4			
ADAM1A		Homozygous null mice display male infertility with asthenozoospermia.	
AS3MT		Mice homozygous for a null allele have abnormalities in arsenic methylation and in the distribution/retention of orally administered arsenate	
ATP2B1		Homozygous null mice display embryonic lethality	
ATXN2	Spinocerebellar ataxia 2 (SCA2) [MIM:183090]	Homozygous mice exhibit an enlarged fat pad, hepatic steatosis and enlarged seminal vesicles. A mild defect in motor learning is seen, but no other notable behavioral or neurological defects are detectable	Spinocerebellar Ataxia 2; SCA2 OMIM: 183090
C10orf107			
C10orf32			
C15orf17			
CLCN6		Mice homozygous for a knock-out allele exhibit impaired nociception, mild behavioral abnormalities, and a progressive neuropathy of the central and peripheral nervous systems with features of neuronal ceroid lipofuscinosis (a lysosomal storage disease).	Ceroid Lipofuscinosis, Neuronal, 3; CLN3 OMIM: 204200
CNNM2	Hypomagnesemia 6 (HOMG6)		
COX5A			
CPLX3		Mice homozygous for a null allele are fertile, viable and exhibit normal synaptic transmission	
CSK		Homozygotes for targeted null mutations exhibit growth retardation, neural tube defects, and developmental arrest at the 10-12 somite stage. Mutants die between embryonic days nine and ten.	
CUX2		Homozygotes for a targeted null mutation exhibit various neural defects.	
CYP17A1	Adrenal hyperplasia 5 (AH5) [MIM:202110]	Homozygous null embryos display early embryonic lethality.	
CYP1A2		Mice homozygous for a null allele display resitance to some signs of TCDD induced toxicity but do not display any gross abnormalities in the abscence of treatment.	
FAM109A			
FES		Homozygotes for a null allele show partial in utero lethality, runting, altered hematopoietic homeostasis and macrophage function, skin lesions and susceptibility to bacterial infection. Homozygotes for another null allele show enhanced LPS sensitivity, altered hematopoiesis and larger litter size.	
FGF5		Mutations in this gene result in significantly longer pelage hair.	

FURIN		Homozygous null embryos die at E10.5-E11.5. Embryos homozygous for one knock-out allele show multiple tissue abnormalities including abnormal yolk sac vasculature and chorioallantoic fusion, failure of axial rotation, a kinked neural tube, exencephaly and severe ventral closure and cardiac defects.	
HECTD4			
HFE	Hemochromatosis 1 (HFE1) [MIM:235200];Variegate porphyria (VP) [MIM:176200];Microvascular complications of	Mutation of this gene affects iron metabolism. Homozygotes for targeted null mutations exhibit increased intestinal iron absorption and an elevated hepatic iron load but reduced duodenal iron stores. Heterozygotes also accumulate more iron than normal.	Hemochromatosis, Type 1; HFE1 235200
HIST1H1T		Homozygous null mice develop normally and exhibit normal testicular morphology, spermatogenesis and fertility.	
HIST1H4C			
ID1		Homozygotes for knockout alleles of both Id1 and Id3 exhibit vascular malformations in the forebrain, lack of vascular branching and sprouting in the neuroectoderm, and impaired angiogenesis in transplanted and spontaneous tumors.	
LMAN1L			
MAPKAPK5		Homozygous mutant mice are viable, fertile, and show no overt abnormalities.	
MIR3193			
MIR4513			
MPI	Congenital disorder of glycosylation 1B (CDG1B) [MIM:602579]	Homozygous null mice display embryonic lethality during organogenesis, variable abnormalities of the yolk sac and embryonic vasculature, and partial penetrance of abnormal chorioallantoic fusion, placental defects, impaired emrbyo turning, increased apoptosis, and posterior axial truncations.	
MTHFR	Methylenetetrahydrofolate reductase deficiency (MTHFRD) [MIM:236250];Ischemic stroke (ISCHSTR) [MIM:601367];Folate-sensitive neural tube defects (FS-NTD) [MIM:601634]	Mice homozygous for disruptions in this gene have elevated plasma levels of homocysteine. They also display delayed growth and development and a reduced survival rate.	Human Disease OMIM ID<br>Homocysteinemia 603174<br>Neural Tube Defects, Folate-Sensitive 601634
NAA25			
NPPA	Atrial fibrillation, familial, 6 (ATFB6) [MIM:612201];	Homozygotes are chronically hypertensive partly due to changes in peripheral resistance and increased central AT1-receptor activation, and show salt-sensitive hypertension and abnormal pulmonary vascular remodeling with increased ventricular mass and muscularization of peripheral pulmonary vessels.	
NT5C2			
PLCD3			
PLEKHA7			

PTPN11	LEOPARD syndrome 1 (LEOPARD1) [MIM:151100];Noonan syndrome 1 (NS1) [MIM:163950];Leukemia, juvenile myelomonocytic (JMML) [MIM:607785];Metachondromatosis (MC) [MIM:156250]	Homozygous null mutants exhibit abnormal mesoderm patterning leading to a failure of gastrulation and death by embryonic day 10.5. In heterozygous state the null mutant acts as a dominant enhancer of a mild epidermal growth factor receptor mutation.	Human Disease OMIM ID<br>Juvenile Myelomonocytic Leukemia; JMML 607785<br>Leopard Syndrome 1; LPRD1 151100<br>Noonan Syndrome 1; NS1 163950
SCAMP2			
SH2B3	Celiac disease 13 (CELIAC13) [MIM:612011];Diabetes mellitus, insulin-dependent (IDDM) [MIM:222100]	Mice homozygous for a knock-out allele exhibit severe perturbations in hematopoiesis, splenomegaly, and abnormal lymphoid and myeloid homeostasis. Mice homozygous for a different knock-out allele display altered mobility of hematopoietic stem/progenitor cells.	
TRAFD1		Mice homozygous for a null allele exhibit increased susceptibility to endotoxin shock and decreased susceptibility to viral infection.	
ULK3			
WBP1L			

Table S3 Score construction for BP-associated genes

Gene_Symbol	P_GRAIL<0.05	GRAIL candidate	HuGE	OMIM	MGI	STRING	DAPPLE	DAVID	CAD association	Score
ACAD10	0	0	1	0	0	0	0	0	0	1
ACBD4	0	0	0	0	0	1	0	0	0	1
ADAM1A	0	0	1	0	1	1	0	0	0	3
AS3MT	0	0	1	0	1	1	0	0	1	4
ATP2B1	0	1	1	0	1	1	1	0	0	5
ATXN2	0	0	1	1	1	1	1	1	1	7
C10orf107	0	0	1	0	0	1	0	0	0	2
C10orf32	0	0	0	0	0	1	0	0	1	2
C15orf17	0	0	0	0	0	0	0	0	0	0
CLCN6	1	0	1	0	1	1	0	1	0	5
CNNM2	0	0	1	1	0	1	0	0	1	4
COX5A	1	0	0	0	0	1	1	1	0	4
CPLX3	1	1	1	0	1	1	0	0	0	5
CSK	0	0	1	0	1	1	1	0	0	4
CUX2	0	0	1	0	1	1	0	0	1	4
CYP17A1	1	0	1	1	1	1	1	1	1	8
CYP1A2	0	0	1	0	1	1	1	1	0	5
FAM109A	0	0	0	0	0	1	0	0	1	2
FES	0	1	1	0	1	1	1	0	0	5
FGF5	0	1	1	0	1	1	1	0	0	5
FURIN	0	0	1	0	1	1	1	1	1	6
HECTD4	1	0	1	0	0	1	0	0	1	4
HFE	0	0	1	3	1	1	0	1	0	7
HIST1H1T	0	0	1	0	1	1	0	0	0	3
HIST1H4C	0	0	1	0	0	1	1	0	0	3
ID1	1	0	0	0	1	1	1	1	0	5
LMAN1L	0	0	1	0	0	1	0	0	0	2
MAPKAPK5	0	0	0	0	1	0	1	0	1	3
MIR3193	0	0	0	0	0	1	0	0	0	1
MIR4513	0	0	0	0	0	1	0	0	0	1
MPI	0	0	0	1	1	1	1	0	0	4
MTHFR	0	0	1	3	1	1	0	1	0	7
NAA25	0	0	1	0	0	1	0	0	1	3
NPPA	1	1	1	1	1	1	1	1	0	8
NT5C2	0	0	1	0	0	1	1	0	1	4
PLCD3	1	1	1	0	0	1	0	0	0	4
PLEKHA7	0	1	1	0	0	1	0	0	0	3
PTPN11	0	0	1	4	1	1	1	1	1	10
SCAMP2	0	0	0	0	0	1	1	1	0	3
SH2B3	0	0	1	2	1	1	1	0	1	7
TRAFD1	0	0	0	0	1	1	1	0	1	4
ULK3	0	0	1	0	0	1	1	0	0	3
WBP1L	0	0	1	0	0	1	0	0	0	2
