# Supplementary figures and images for: Novel Genes Affecting Blood Pressure Detected Via Gene-Based Association Analysis
Source: G3 (Bethesda). 2015 Mar 26;5(6):1035–42. doi: 10.1534/g3.115.016915 (PMC4478534; doi:10.1534/g3.115.016915)

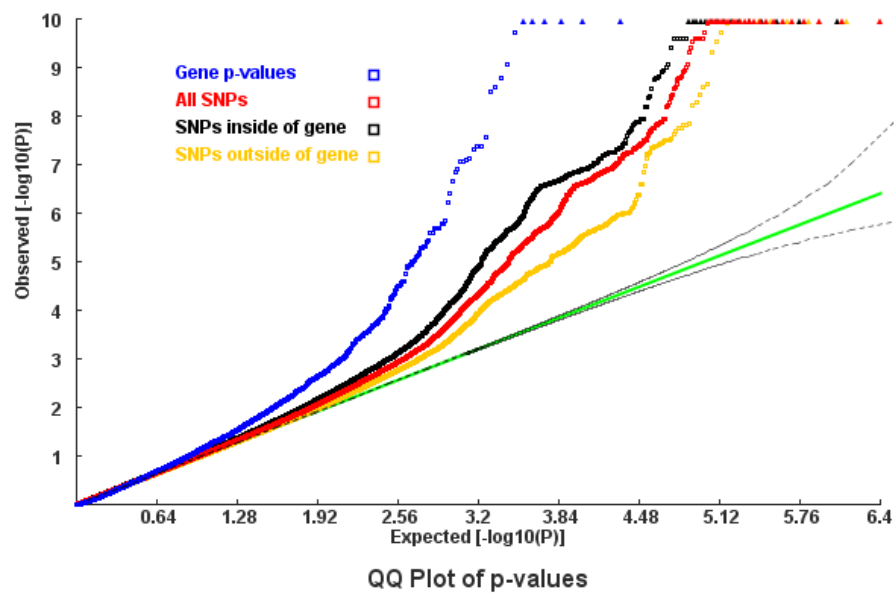

Figure S1 QQ plot of genes and SNPs for DBP (ICBP GWAS data).

Supplement: Supporting Information [file supp_g3.115.016915_FigureS1.pdf]

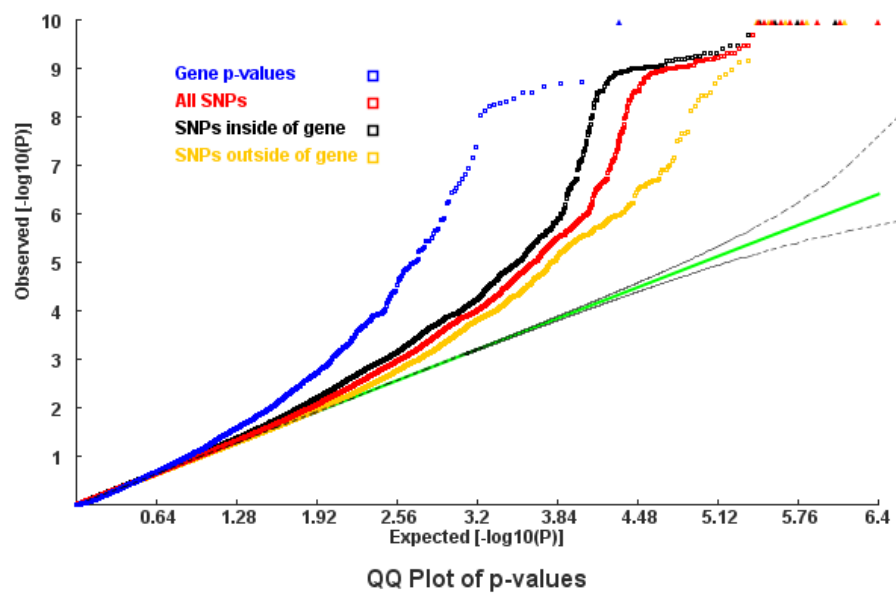

Figure S2 QQ plot of genes and SNPs for SBP (ICBP GWAS data).

Supplement: Supporting Information [file supp_g3.115.016915_FigureS2.pdf]
