# Supplementary material for: Novel Genes Affecting Blood Pressure Detected Via Gene-Based Association Analysis
Source: G3 (Bethesda). 2015 Mar 26;5(6):1035–42. doi: 10.1534/g3.115.016915 (PMC4478534; doi:10.1534/g3.115.016915)
Supplement: Supporting Information [file supp_g3.115.016915_FigureS7.pdf]

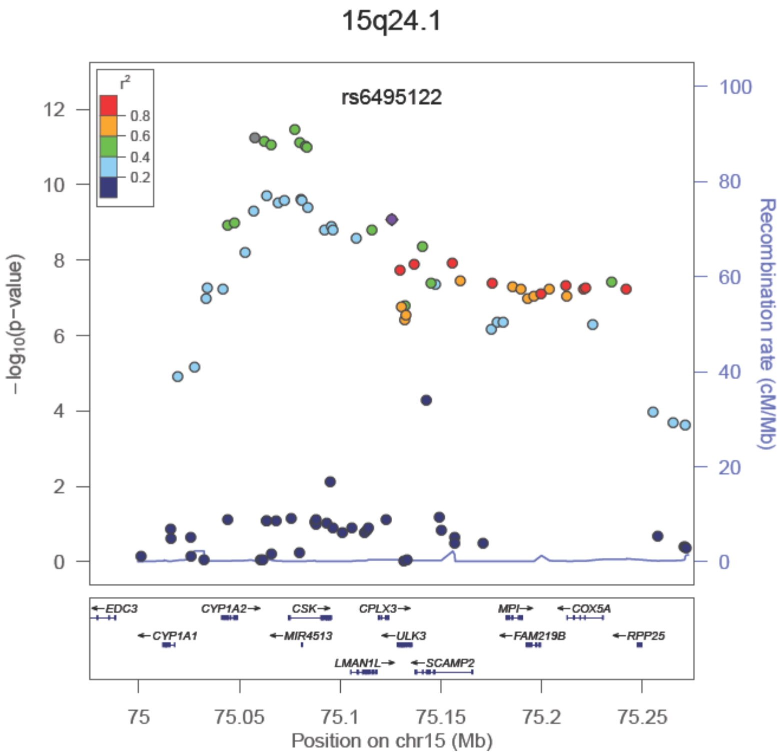

Figure S7 Regional association plot for DBP at 15q24.1 (ICBP GWAS data)

COX5A, *C15orf17*, *MIR4513*, *SCAMP2* were unreported genes in this region with gene-based P value $< 2.3\times10^{-6}$.
