# Supplementary material for: Novel Genes Affecting Blood Pressure Detected Via Gene-Based Association Analysis
Source: G3 (Bethesda). 2015 Mar 26;5(6):1035–42. doi: 10.1534/g3.115.016915 (PMC4478534; doi:10.1534/g3.115.016915)
Supplement: Supporting Information [file supp_g3.115.016915_FigureS8.pdf]

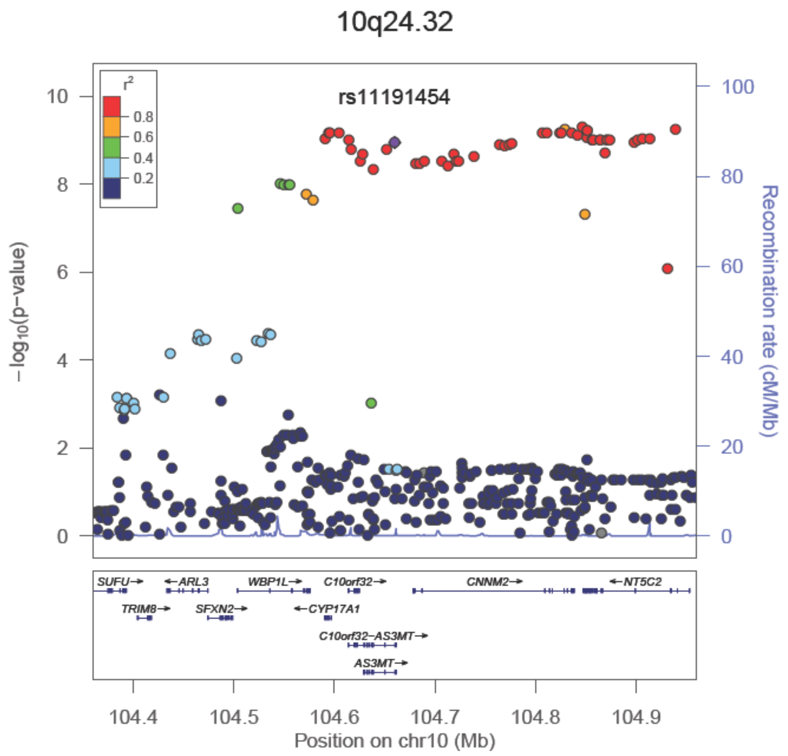

Figure S8 Regional association plot for SBP at 10q24.32 (ICBP GWAS data)

C10orf32, *C10orf26* (*WBP1L*) were unreported genes in this region with gene-based P value < 2.3×10^{-6}.
