# Supplementary material for: Novel Genes Affecting Blood Pressure Detected Via Gene-Based Association Analysis
Source: G3 (Bethesda). 2015 Mar 26;5(6):1035–42. doi: 10.1534/g3.115.016915 (PMC4478534; doi:10.1534/g3.115.016915)
Supplement: Supporting Information [file supp_g3.115.016915_TableS1.pdf]

Table S1 Information for the BP-associated genes collected from the HuGE Navigator

Gene_Symbol	Abortion	AIDS	Adenocarcinoma	Alcoholism	Alzheimer Disease	Amyotrophic Lateral Sclerosis	Anemia	Arsenic Poisoning Phenopedia	Arthritis, Rheumatoid	Bipolar Disorder	Breast Neoplasms	Cardiovascular Diseases	Celiac Disease	cocaine dependence	Colorectal Neoplasms	COPD	Crohn Disease	Diabetes Mellitus, Type 1	Diabetes Mellitus, Type 2
ACAD10		Y									Y								Y
ACBD4																			
ADAM1A																		Y	
AS3MT								Y				Y						Y	
ATP2B1												Y							Y
ATXN2						Y						Y							
C10orf107					Y							Y							Y
C10orf32																			
C15orf17																			
CLCN6												Y							Y
CNNM2					Y							Y							
COX5A																			
CPLX3														Y					
CSK												Y							
CUX2																	Y	Y	Y
CYP17A1			Y		Y						Y	Y							
CYP1A2			Y								Y	Y			Y	Y			
FAM109A																			
FES												Y							
FGF5				Y								Y							
FURIN												Y							
HECTD4																			Y
HFE					Y	Y						Y							Y
HIST1H1T	Y																		
HIST1H4C	Y																		
ID1																			
LMAN1L																			
MAPKAPK5																			
MIR3193																			
MIR4513																			
MPI																			
MTHFR	Y			Y	Y		Y		Y		Y	Y			Y		Y		Y
NAA25									Y	Y		Y					Y	Y	Y
NPPA												Y							Y
NT5C2												Y							
PLCD3																			
PLEKHA7																			
PTPN11																			
SCAMP2																			
SH2B3												Y	Y					Y	
TRAFD1																			
ULK3																			
WBP1L					Y														

Gene_Symbol	Diabetic Nephropathies	Disease Progression	Down Syndrome	Dyslipidemias	Endometrial Neoplasms	Folic Acid Deficiency	Gout	Graves' disease	Hemochromatosis	Hepatitis	Hyperhomocysteinemia	Hypertension	hypothyroidism	Insulin Resistance	Iron Overload	Kidney Failure, Chronic	Leukemia	Liver disease	Lung Neoplasms
ACAD10		Y												Y					
ACBD4																			
ADAM1A	Y																		
AS3MT												Y							
ATP2B1				Y								Y							
ATXN2							Y					Y				Y			
C10orf107												Y							
C10orf32																			
C15orf17																			
CLCN6												Y							
CNNM2												Y							
COX5A																			
CPLX3																			
CSK												Y							
CUX2																			
CYP17A1					Y							Y							Y
CYP1A2					Y														Y
FAM109A																			
FES												Y				Y			
FGF5												Y							
FURIN												Y				Y			
HECTD4												Y							
HFE									Y	Y		Y			Y			Y	
HIST1H1T																			
HIST1H4C																			
ID1																			
LMAN1L																			
MAPKAPK5																			
MIR3193																			
MIR4513																			
MPI																			
MTHFR	Y		Y	Y		Y					Y	Y		Y		Y		Y	Y
NAA25																			
NPPA												Y							
NT5C2												Y							
PLCD3												Y							
PLEKHA7												Y							
PTPN11																	Y		
SCAMP2																			
SH2B3								Y		Y		Y	Y						
TRAFD1																			
ULK3												Y							
WBP1L																			

Gene_Symbol	Metabolic Diseases	Multiple Sclerosis	Neural Tube Defects	Noonan Syndrome	Obesity	Pancreatic Neoplasms	Parkinson Disease	Prostatic Neoplasms	Schizophrenia	Spinocerebellar Ataxias	Stroke	Thrombosis	Tobacco Use Disorder	Urinary Bladder Neoplasms	Vitiligo
ACAD10													Y		
ACBD4															
ADAM1A															
AS3MT									Y					Y	
ATP2B1	Y				Y										
ATXN2						Y	Y			Y					
C10orf107													Y		
C10orf32															
C15orf17															
CLCN6											Y		Y		
CNNM2									Y		Y		Y		
COX5A															
CPLX3															
CSK					Y										
CUX2															
CYP17A1					Y			Y			Y		Y		
CYP1A2							Y	Y	Y					Y	
FAM109A															
FES											Y				
FGF5															
FURIN											Y				
HECTD4	Y												Y		
HFE		Y													
HIST1H1T															
HIST1H4C															
ID1															
LMAN1L													Y		
MAPKAPK5															
MIR3193															
MIR4513															
MPI															
MTHFR	Y		Y				Y		Y		Y	Y		Y	
NAA25															
NPPA											Y				
NT5C2									Y		Y				
PLCD3															
PLEKHA7															
PTPN11				Y											
SCAMP2															
SH2B3		Y									Y				Y
TRAFD1															
ULK3							Y								
WBP1L															
