# Supplementary material for: Novel Genes Affecting Blood Pressure Detected Via Gene-Based Association Analysis
Source: G3 (Bethesda). 2015 Mar 26;5(6):1035–42. doi: 10.1534/g3.115.016915 (PMC4478534; doi:10.1534/g3.115.016915)
Supplement: Supporting Information [file supp_g3.115.016915_TableS2.pdf]

Table S2 Information of monogenic syndromes and mouse models for BP-associated genes

Gene_Symbol	OMIM	MGI	Human Diseases Modeled in Mice
ACAD10			
ACBD4			
ADAM1A		Homozygous null mice display male infertility with asthenozoospermia.	
AS3MT		Mice homozygous for a null allele have abnormalities in arsenic methylation and in the distribution/retention of orally administered arsenate	
ATP2B1		Homozygous null mice display embryonic lethality	
ATXN2	Spinocerebellar ataxia 2 (SCA2) [MIM:183090]	Homozygous mice exhibit an enlarged fat pad, hepatic steatosis and enlarged seminal vesicles. A mild defect in motor learning is seen, but no other notable behavioral or neurological defects are detectable	Spinocerebellar Ataxia 2; SCA2 OMIM: 183090
C10orf107			
C10orf32			
C15orf17			
CLCN6		Mice homozygous for a knock-out allele exhibit impaired nociception, mild behavioral abnormalities, and a progressive neuropathy of the central and peripheral nervous systems with features of neuronal ceroid lipofuscinosis (a lysosomal storage disease).	Ceroid Lipofuscinosis, Neuronal, 3; CLN3 OMIM: 204200
CNNM2	Hypomagnesemia 6 (HOMG6)		
COX5A			
CPLX3		Mice homozygous for a null allele are fertile, viable and exhibit normal synaptic transmission	
CSK		Homozygotes for targeted null mutations exhibit growth retardation, neural tube defects, and developmental arrest at the 10-12 somite stage. Mutants die between embryonic days nine and ten.	
CUX2		Homozygotes for a targeted null mutation exhibit various neural defects.	
CYP17A1	Adrenal hyperplasia 5 (AH5) [MIM:202110]	Homozygous null embryos display early embryonic lethality.	
CYP1A2		Mice homozygous for a null allele display resitance to some signs of TCDD induced toxicity but do not display any gross abnormalities in the abscence of treatment.	
FAM109A			
FES		Homozygotes for a null allele show partial in utero lethality, runting, altered hematopoietic homeostasis and macrophage function, skin lesions and susceptibility to bacterial infection. Homozygotes for another null allele show enhanced LPS sensitivity, altered hematopoiesis and larger litter size.	
FGF5		Mutations in this gene result in significantly longer pelage hair.	

FURIN		Homozygous null embryos die at E10.5-E11.5. Embryos homozygous for one knock-out allele show multiple tissue abnormalities including abnormal yolk sac vasculature and chorioallantoic fusion, failure of axial rotation, a kinked neural tube, exencephaly and severe ventral closure and cardiac defects.	
HECTD4			
HFE	Hemochromatosis 1 (HFE1) [MIM:235200];Variegate porphyria (VP) [MIM:176200];Microvascular complications of	Mutation of this gene affects iron metabolism. Homozygotes for targeted null mutations exhibit increased intestinal iron absorption and an elevated hepatic iron load but reduced duodenal iron stores. Heterozygotes also accumulate more iron than normal.	Hemochromatosis, Type 1; HFE1 235200
HIST1H1T		Homozygous null mice develop normally and exhibit normal testicular morphology, spermatogenesis and fertility.	
HIST1H4C			
ID1		Homozygotes for knockout alleles of both Id1 and Id3 exhibit vascular malformations in the forebrain, lack of vascular branching and sprouting in the neuroectoderm, and impaired angiogenesis in transplanted and spontaneous tumors.	
LMAN1L			
MAPKAPK5		Homozygous mutant mice are viable, fertile, and show no overt abnormalities.	
MIR3193			
MIR4513			
MPI	Congenital disorder of glycosylation 1B (CDG1B) [MIM:602579]	Homozygous null mice display embryonic lethality during organogenesis, variable abnormalities of the yolk sac and embryonic vasculature, and partial penetrance of abnormal chorioallantoic fusion, placental defects, impaired emrbyo turning, increased apoptosis, and posterior axial truncations.	
MTHFR	Methylenetetrahydrofolate reductase deficiency (MTHFRD) [MIM:236250];Ischemic stroke (ISCHSTR) [MIM:601367];Folate-sensitive neural tube defects (FS-NTD) [MIM:601634]	Mice homozygous for disruptions in this gene have elevated plasma levels of homocysteine. They also display delayed growth and development and a reduced survival rate.	Human Disease OMIM ID<br>Homocysteinemia 603174<br>Neural Tube Defects, Folate-Sensitive 601634
NAA25			
NPPA	Atrial fibrillation, familial, 6 (ATFB6) [MIM:612201];	Homozygotes are chronically hypertensive partly due to changes in peripheral resistance and increased central AT1-receptor activation, and show salt-sensitive hypertension and abnormal pulmonary vascular remodeling with increased ventricular mass and muscularization of peripheral pulmonary vessels.	
NT5C2			
PLCD3			
PLEKHA7			

PTPN11	LEOPARD syndrome 1 (LEOPARD1) [MIM:151100];Noonan syndrome 1 (NS1) [MIM:163950];Leukemia, juvenile myelomonocytic (JMML) [MIM:607785];Metachondromatosis (MC) [MIM:156250]	Homozygous null mutants exhibit abnormal mesoderm patterning leading to a failure of gastrulation and death by embryonic day 10.5. In heterozygous state the null mutant acts as a dominant enhancer of a mild epidermal growth factor receptor mutation.	Human Disease OMIM ID Juvenile Myelomonocytic Leukemia; JMML 607785 Leopard Syndrome 1; LPRD1 151100 Noonan Syndrome 1; NS1 163950
SCAMP2			
SH2B3	Celiac disease 13 (CELIAC13) [MIM:612011];Diabetes mellitus, insulin-dependent (IDDM) [MIM:222100]	Mice homozygous for a knock-out allele exhibit severe perturbations in hematopoiesis, splenomegaly, and abnormal lymphoid and myeloid homeostasis. Mice homozygous for a different knock-out allele display altered mobility of hematopoietic stem/progenitor cells.	
TRAFD1		Mice homozygous for a null allele exhibit increased susceptibility to endotoxin shock and decreased susceptibility to viral infection.	
ULK3			
WBP1L			
