# Supplementary material for: Novel Genes Affecting Blood Pressure Detected Via Gene-Based Association Analysis
Source: G3 (Bethesda). 2015 Mar 26;5(6):1035–42. doi: 10.1534/g3.115.016915 (PMC4478534; doi:10.1534/g3.115.016915)
Supplement: Supporting Information [file supp_g3.115.016915_TableS3.pdf]

Table S3 Score construction for BP-associated genes

Gene_Symbol	P_GRAIL<0.05	GRAIL candidate	HuGE	OMIM	MGI	STRING	DAPPLE	DAVID	CAD association	Score
ACAD10	0	0	1	0	0	0	0	0	0	1
ACBD4	0	0	0	0	0	1	0	0	0	1
ADAM1A	0	0	1	0	1	1	0	0	0	3
AS3MT	0	0	1	0	1	1	0	0	1	4
ATP2B1	0	1	1	0	1	1	1	0	0	5
ATXN2	0	0	1	1	1	1	1	1	1	7
C10orf107	0	0	1	0	0	1	0	0	0	2
C10orf32	0	0	0	0	0	1	0	0	1	2
C15orf17	0	0	0	0	0	0	0	0	0	0
CLCN6	1	0	1	0	1	1	0	1	0	5
CNNM2	0	0	1	1	0	1	0	0	1	4
COX5A	1	0	0	0	0	1	1	1	0	4
CPLX3	1	1	1	0	1	1	0	0	0	5
CSK	0	0	1	0	1	1	1	0	0	4
CUX2	0	0	1	0	1	1	0	0	1	4
CYP17A1	1	0	1	1	1	1	1	1	1	8
CYP1A2	0	0	1	0	1	1	1	1	0	5
FAM109A	0	0	0	0	0	1	0	0	1	2
FES	0	1	1	0	1	1	1	0	0	5
FGF5	0	1	1	0	1	1	1	0	0	5
FURIN	0	0	1	0	1	1	1	1	1	6
HECTD4	1	0	1	0	0	1	0	0	1	4
HFE	0	0	1	3	1	1	0	1	0	7
HIST1H1T	0	0	1	0	1	1	0	0	0	3
HIST1H4C	0	0	1	0	0	1	1	0	0	3
ID1	1	0	0	0	1	1	1	1	0	5
LMAN1L	0	0	1	0	0	1	0	0	0	2
MAPKAPK5	0	0	0	0	1	0	1	0	1	3
MIR3193	0	0	0	0	0	1	0	0	0	1
MIR4513	0	0	0	0	0	1	0	0	0	1
MPI	0	0	0	1	1	1	1	0	0	4
MTHFR	0	0	1	3	1	1	0	1	0	7
NAA25	0	0	1	0	0	1	0	0	1	3
NPPA	1	1	1	1	1	1	1	1	0	8
NT5C2	0	0	1	0	0	1	1	0	1	4
PLCD3	1	1	1	0	0	1	0	0	0	4
PLEKHA7	0	1	1	0	0	1	0	0	0	3
PTPN11	0	0	1	4	1	1	1	1	1	10
SCAMP2	0	0	0	0	0	1	1	1	0	3
SH2B3	0	0	1	2	1	1	1	0	1	7
TRAFD1	0	0	0	0	1	1	1	0	1	4
ULK3	0	0	1	0	0	1	1	0	0	3
WBP1L	0	0	1	0	0	1	0	0	0	2
